# Stability of SARS-CoV-2 on Commercial Aircraft Interior Surfaces with Implications for Effective Control Measures

**DOI:** 10.3390/ijerph20166598

**Published:** 2023-08-18

**Authors:** Kenrie P. Y. Hui, Alex W. H. Chin, John Ehret, Ka-Chun Ng, Malik Peiris, Leo L. M. Poon, Karen H. M. Wong, Michael C. W. Chan, Ian Hosegood, John M. Nicholls

**Affiliations:** 1 School of Public Health, Li Ka Shing Faculty of Medicine, The University of Hong Kong, Pok Fu Lam HKG, Hong Kong SAR, China; kenrie@hku.hk (K.P.Y.H.);; 2 Centre for Immunology & Infection, Hong Kong Science Park HKG, Hong Kong SAR, China; 3 Qantas Airways Ltd., Qantas 10 Bourke Rd Mascot, Sydney, NSW 2020, Australia; 4 Electron Microscopy Unit, Queen Mary Hospital, The University of Hong Kong, Pok Fu Lam HKG, Hong Kong SAR, China; 5 Department of Pathology, School of Clinical Medicine, Li Ka Shing Faculty of Medicine, The University of Hong Kong, Queen Mary Hospital, Pok Fu Lam HKG, Hong Kong SAR, China

**Keywords:** SARS-CoV-2, transmission, fomite

## Abstract

Background: The COVID-19 pandemic from 2019 to 2022 devastated many aspects of life and the economy, with the commercial aviation industry being no exception. One of the major concerns during the pandemic was the degree to which the internal aircraft environment contributed to virus transmission between humans and, in particular, the stability of SARS-CoV-2 on contact surfaces in the aircraft cabin interior. Method: In this study, the stability of various major strains of SARS-CoV-2 on interior aircraft surfaces was evaluated using the TCID_50_ assessment. Results: In contrast to terrestrial materials, SARS-CoV-2 was naturally less stable on common contact points in the aircraft interior, and, over a 4 h time period, there was a 90% reduction in culturable virus. Antiviral and surface coatings were extremely effective at mitigating the persistence of the virus on surfaces; however, their benefit was diminished by regular cleaning and were ineffective after 56 days of regular use and cleaning. Finally, successive strains of SARS-CoV-2 have not evolved to be more resilient to survival on aircraft surfaces. Conclusions: We conclude that the mitigation strategies for SARS-CoV-2 on interior aircraft surfaces are more than sufficient, and epidemiological evidence over the past three years has not found that surface spread is a major route of transmission.

## 1. Introduction

The SARS-CoV-2 virus emerged in China in late 2019 and within 3 months spread to most countries in the world. At the end of November 2019, cases of pneumonia attributed to a novel coronavirus were described as occurring in the proximity of a live animal and seafood market in the city of Wuhan, Hubei Province, China. These cases were reported to the World Health Organization on 31 December 2019. In Hong Kong, Macao, and Taiwan there was a concern that this could be a repeat of the 2003 SARS outbreak. Of the 44 cases reported, 11 were severely ill while the remaining 33 patients were in stable condition [1].

It is still undermined if the first infections of humans by SARS-CoV-2 arose from an animal source or was due to a “leak” from the Wuhan Scientific Biosafety Laboratory [2]. Three years later, it is now acknowledged that SARS-CoV-2 is endemic, and thus unlikely to be eliminated. Clinical and post-mortem studies have concluded that SARS-CoV-2 is primarily a respiratory virus, with replication occurring in the upper respiratory tract [3] and limited systemic distribution throughout the body. Laboratory studies using hamsters and transgenic mice together with extensive epidemiological studies have concluded that the initial site of replication is in the upper respiratory tract, with entry through the nasal and oral cavities. During the early stages of the pandemic, there was considerable attention on the olfactory mucosa as the initial site of replication, in particular, the sustentacular cells, as anosmia was a common presenting symptom. The conclusion from large epidemiological studies is that the main routes of transmission are by air large droplets and smaller aerosols, with limited data on physical and fomite transmission [4].

The ocular route for the transmission of SARS-CoV-2 has also been investigated, and though some patients reported symptoms such as folliculitis or keratoconjunctivitis, this has not been a major feature of infection with Omicron or later strains compared to the ancestral strain (11% versus 31%) [5]. Even though many review studies concluded that the risk of ocular transmission is low, many professional bodies advocate the use of goggles and face shields in high-risk settings.

In the early stages of the 2020 outbreak, there was concern over the fecal—oral route of transmission, as viral RNA was detected in the stools of infected patients (similar to that documented in the 2003 SARS outbreak) [6,7]. Epidemiological studies performed in 2003 showed the transmission of SARS-CoV via wastewater systems in old residential blocks. Indeed, wastewater surveillance has been a cornerstone of assessing the spread of all strains of SARS-CoV-2 at the community level [8], as well as in aircraft wastewater [7], and thus has been advocated as an early warning system for future pandemics [9]. Though these studies using PCR have been invaluable in monitoring the disease burden, there is little published evidence of culturable viruses in wastewater systems, and, thus, unlike viruses such as the norovirus which have clearly documented fecal—oral routes of transmission, comprehensive reviews [10,11] have concluded that there are limited published data to show that the infectious virus leading to transmission via this route has been a major factor contributing to the global spread of the virus.

In order to investigate the role of fomites or surfaces in the transmission of SARS-CoV-2, a number of laboratory-based studies were performed in early 2020 [12] which showed that, after the inoculation of a number of terrestrial surfaces, the virus could be cultured for periods up to 7 days, depending on the type of material.

In early to mid-2020, there were sporadic reports of passengers from the same flight testing positive for SARS-CoV-2 after arrival or in quarantine (reviewed in [13,14]), conceding that passenger flights were at risk for transmission, though it was acknowledged there were many factors determining the risk of air travel-related transmission including length of flight, movement of passengers, seat occupancy, and interactions outside the aircraft [15,16,17].

Aircraft interiors were considered high-risk areas for a number of reasons. Firstly, they had a high occupancy density and, for operational reasons, the interior was kept at relatively low humidity (<15%) [18], which in the experimental setting, potentially favored aerosol transmission by droplet nuclei as the low humidity allowed desiccation into smaller aerosols that are able to travel further in the environment [19], and, being smaller, were more likely to infect the lower respiratory tract (including the bronchi and alveoli) compared to the upper nasal tract [20]. Secondly, with the high passenger density, there was clustering in galley areas and around restrooms, favoring aerosol transmission, and this was more evident on long-distance flights [14]. The role of masks and passenger density in transmission was investigated in a number of epidemiological studies [21], coming to the conclusion that the risks were higher for passengers within three rows of an index patient [22,23].

During early 2020, particular attention was given to the commercial aviation industry, as historical data from the 2003 SARS outbreak showed that transmission could occur in the aircraft interior [24,25]. Indeed, early in the SARS-CoV-2 pandemic, there was documented evidence of transmission in the aircraft environment (reviewed in [13,14,22,26,27]), with most studies concluding that the primary role of transmission was via the aerosol route.

A number of strategies to mitigate aerosol transmission in the aircraft setting were proposed and utilized in 2020 during the early stages of the pandemic, including leaving the middle seat empty, serving meals at different times to reduce “mask off times”, the use of adjustable over seat vents, boarding procedures, etc. (reviewed in [15,17,27,28,29]), however, a number of review studies raised the important point that the transmission risks were not just in flight but could occur during planing, deplaning, or at customs/immigration checkpoints, and that most published studies were retrospective with low-quality evidence [16]. Since considerable manpower and financial resources were spent on surface transmission mitigation strategies, a laboratory investigation on SARS-CoV-2 survival on aircraft interior surfaces was considered an area worthy of investigation.

## 2. Materials and Methods

### 2.1. SARS-CoV-2 Isolation

Vero E6 (E6) cells were used for virus isolation and propagation of the wild-type virus (hCoV-19/Hong Kong/WHV-HK61-P3/2020, Genbank accession ID: OM403304) and Vero E6-TMPRSS2 (T2) overexpressed cells were used for the Delta (hCoV-19/Hong Kong/VOC0013P2D5/2021, Genbank accession ID: OM403308) and Omicron variants (BA.2.3, SARS-CoV-2/human/HKG/Consensus_VOC-588-P3-S18-iseq/2022, Genbak accession ID: ON026861; BA.5, SARS-CoV-2/human/USA/COR-22-063113/2022, Genbank accession ID: ON972631). Both cell lines were cultured in DMEM with 10% FBS, and the clinical samples were collected from patients from January 2020 to July 2022 and isolated as previously described [30]. Viruses were isolated from clinical specimens of the throat and nasopharyngeal swabs from patients infected with SARS-CoV-2 stored in a virus transport medium. Vero E6-TMPRSS2 (T2) overexpressed cells were seeded in a 24-well plate at a cell density of 1 × 10^5^ per well. The cells were inoculated with 50 ul of sample and 1 mL of DMEM medium supplemented with 2% Fetal Bovine Serum (FBS) was added. After 1 h of incubation at 37 °C, the cells were washed once with PBS, replenished with fresh medium, and the cytopathic effect (CPE) was monitored daily. Culture supernatants were harvested once the CPE reached around 50% and the virus stock was defined as passage 1. Further virus propagation was performed in corresponding E6 or T2 cells.

The identity of the virus strain was determined by full genome sequencing [30]. We deduced near full-length genomes and variants from the samples with the iSeq 100 System (Illumina, San Diego, CA, USA) using a sequencing protocol previously described by us. Briefly, the virus genome was reverse transcribed with multiple gene-specific primers targeting different regions of the viral genome. The synthesized cDNA was then subjected to multiple overlapping 2-kb PCRs for full-genome amplification. PCR amplicons obtained from the same specimen were pooled and sequenced using the Novaseq or iSeq sequencing platform (Illumina, San Diego, CA, USA). The sequencing library was prepared by Nextera XT (Illumina, San Diego, CA, USA).

The virus stock was aliquoted and stored frozen at −80 °C. Aliquots were titrated to determine the plaque forming unit (pfu) in respective E6 or T2 cells. The experiments were carried out in a Bio-safety level 3 (BSL-3) facility at the School of Public Health, Li Ka Shing Faculty of Medicine, The University of Hong Kong.

### 2.2. Aircraft Parts

Interior aircraft components were sourced by the Engineering Division of QANTAS Airways, either from unserviceable components or new ex-factory stock. Each component was sectioned into 1 cm^2^ squares and shipped to Hong Kong for surface testing and inoculation. Each component was sectioned into 30 or more pieces which would allow for multiple evaluations of virus stability as well as chemical analysis. Three pieces of each component were inoculated with each virus. Black tray table samples (approx. 3 mm thickness) from a B737, leather seat covers from an A320, grey tray tables from an A330, thick tray table samples with white foam fill from a Dash 8 Turbo Prop, seat belts/buckles, armrest, window, plastic armrest, and painted aircraft locker latches from a B747, and carpet material from generic aircraft. Video Screen/controller/surrounds were all excess unserviceable parts supplied by Panasonic. Surface treatment and fogging was performed using 0.75% CAS #27668-52-6 (Dimethyloctadecyl [3-(trimethoxysilyl)propyl]ammonium Chloride (40–50 wt.% in methanol). Samples from the Dash 8 Turbo Prop had been cleaned daily with Transclean. A cleaning cycle was defined as an application of ready to use Calla^®^1452 (Zip-Chem, Morgan Hill, CA, USA) followed after 5 min by a single wipe with Transclean (Zep Inc., Atlanta, GA, USA). Multiple cycles of 14 and 28 cleanings were performed on the black tray samples.

### 2.3. SARS-CoV-2 Viability on Surfaces

For the experiment, 1 cm^2^ square pieces of materials with or without treatment were exposed to the SARS-CoV-2 wild-type and variants for 0, 1, 2, and 4 h at 1 × 10^6^ pfu/mL. A total of 5 μL of virus aliquot was added to the surface of the materials and incubated at room temperature. The samples were resuspended in 200 μL of virus transfer medium and were evaluated for infectious viral load by a 50% Tissue Culture Infectious Dose (TCID_50_) assay in Vero E6-TMPRSS2 cells. Three samples were used for each time point for virus stability.

### 2.4. Viral Titration by TCID_50_ Assay

A confluent 96-well tissue culture plate of Vero-E6 or Vero E6-TMPRSS2 cells was prepared one day before the virus titration (TCID_50_) assay. Cells were washed once with PBS and replenished with DMEM (Gibco^TM^, Life Technologies Corporation, New York, NY, USA) with 2% fetal bovine serum (Gibco^TM^, Life Technologies Corporation, New York, NY, USA) supplemented with 100 units/mL penicillin and 100 µg/mL streptomycin (Gibco^TM^, Life Technologies Corporation, New York, NY, USA). Serial dilutions of virus supernatant, from 0.5 log to 7 log, were performed and each virus dilution was added to the plates in quadruplicate. After 4 days of incubation, the plates were observed for cytopathic effect. The end-point of viral dilution leading to CPE in 50% of inoculated wells was estimated using the Karber method [31].

### 2.5. Scanning Electron Microscopy

The surface and morphology of the treated and untreated samples were examined by scanning electron microscopy (SEM, Hitachi S-3400N, Hitachi, Tokyo, Japan), and the elemental compositions were determined by energy-dispersive X-ray spectroscopy (EDS). The percentage concentration of silicon was compared among the treated and untreated samples.

### 2.6. Statistical Analysis

Experiments were performed independently at least two or three times. The results shown in the figures are the calculated mean and standard deviation of the mean. Statistical significance was analyzed using a two-way ANOVA with Bonferroni post-tests for the viral titer (TCID_50_/mL) at various time points and the percentage change of the viral titer. A one-way ANOVA with Bonferroni post-test was used for the silicon concentrations. All the statistical significances were calculated using GraphPad Prism version 9.0. Differences were considered significant at a *p* < 0.05.

## 3. Results

### 3.1. Virus Stability over Time, after Treatment, and Cleaning Cycles Using Ancestral Strain

**Figure 1:**
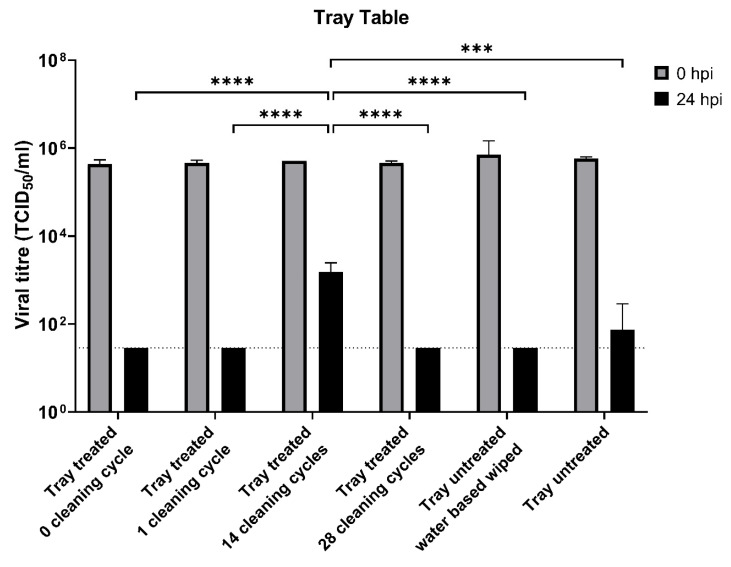
Viral titers of the wild-type SARS-CoV-2 strain on B737-8 tray tables after treatment with compound CAS #27668-52-6 with and without cleaning by standard disinfectants. The horizontal dotted line denotes the limit of detection in the TCID_50_ assay. Data are the geometric mean ± s.d. *n* = 2. Statistical analysis was performed using a two-way ANOVA followed by Bonferroni’s test. *** *p* < 0.001; **** *p* < 0.0001.

Figure 1 shows the viral titer of the wild-type SARS-CoV-2 strain (hCoV-19/Hong Kong/WHV-HK61-P3/2020), 24 h after inoculation of the surface of a tray table from a B737-8. In the untreated sample, after 24 h, there was a three log decrease. The treated tray table showed that at 24 h post-inoculation, there was no viral activity (limit of detection). A single cleaning cycle had a similar effect to the lack of a cleaning cycle, however, after 14 cleaning cycles, there was only a one log decrease. The anti-microbial effect would be similar if a water-based wipe (Transclean, Zep Inc., Atlanta, GA, USA) was used instead of treatment.

### 3.2. Virus Stability over Time on Different Interior Components with and without Treatment

**Figure 2:**
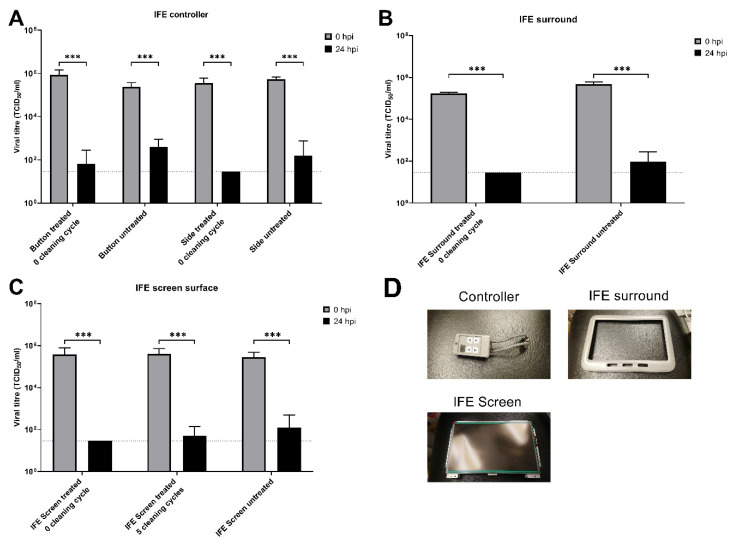
Wild-type virus stability on different contact surfaces 24 h post-exposure, with and without pre-treatment with compound CAS # 27668-52-6. Tested contact surfaces included (**A**) in-flight entertainment (IFE) system controller, (**B**) IFE surround, and (**C**) IFE screen surface. (**D**) Pictures of the samples tested. The horizontal dotted line denotes the limit of detection in the TCID_50_ assay. Data are the geometric mean ±s.d. *n* = 3. Statistical analysis was performed using a two-way ANOVA followed by Bonferroni’s test. *** *p* < 0.001.

The virus survival on different surfaces was then evaluated using the wild-type strain and another common contact site—the in-flight entertainment (IFE) system. Figure 2 shows that 24 h after inoculation, all three areas showed a 3 log decrease in the untreated samples, with treatment being most effective for the screen surface but not the button or the IFE surround.

### 3.3. Delta Strain Virus Stability over Time and on Different Surfaces

**Figure 3:**
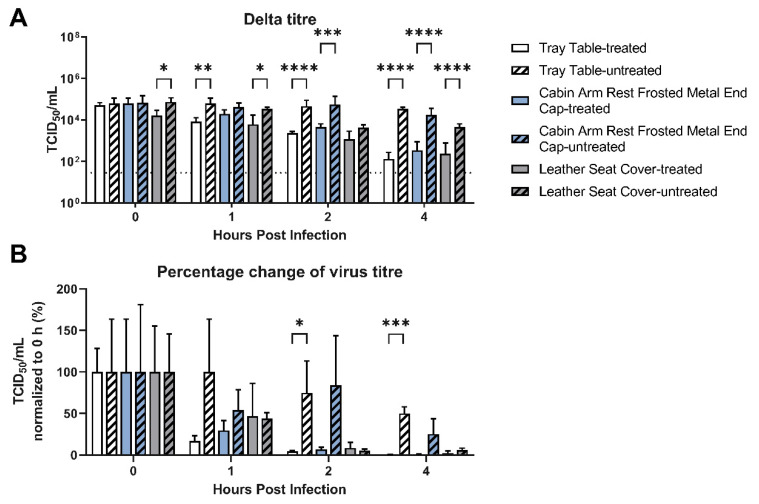
SARS-CoV-2 Delta strain virus stability on different contact surfaces with and without pre-treatment with compound CAS #27668-52-6 presented as titers in (**A**) TCID_50_/mL and (**B**) percentage change of virus titer. The horizontal dotted line denotes the limit of detection in the TCID_50_ assay. Data are the mean ± s.d. *n* = 3. Statistical analysis was performed using a two-way ANOVA followed by Bonferroni’s test. * *p* < 0.05; ** *p* < 0.01; *** *p* < 0.001; and **** *p* < 0.0001.

In 2021, the ancestral strain was replaced by the Delta strain, and we evaluated the virus survival over a shorter time period of 4 h. Figure 3 shows the change within the first 4 h after inoculation with the Delta strain (AY.23, hCoV-19/Hong Kong/VOC0013P2D5/2021). In addition to the tray table, the cabin armrests and leather seat covers were studied. Even without any treatment, by 4 h, there was little remaining culturable virus on the leather seats. The tray table viral titers also dropped by 50%. Similar to Figure 1 with treatment, no virus was able to be cultured after treatment.

### 3.4. Omicron Virus Strain Stability over Time and after Regular Use

**Figure 4:**
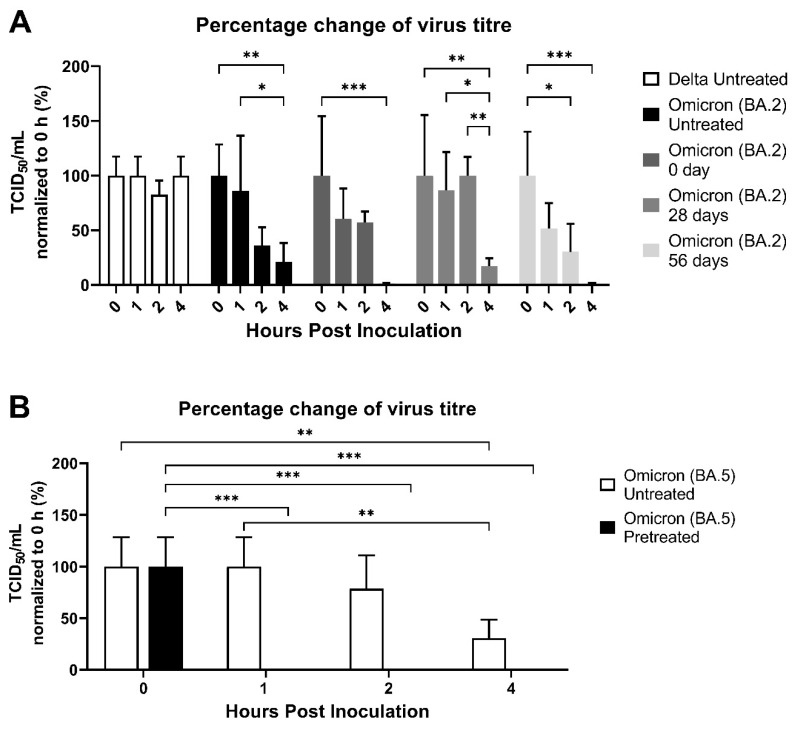
Delta and Omicron virus stability on tray table (Dash 8), with and without pre-treatment with compound CAS #27668-52-6. (**A**) Tables were sampled at 0, 28, and 56 days post-application and regular service. Delta and Omicron (BA.2) virus stability was shown using titers at 0 h as reference. (**B**) Omicron (BA.5) virus stability was assessed on tables in use for 28 days with and without pre-treatment. The percentage change of virus titers was shown using titers at 0 h as reference. Data are the mean ± s.d. *n* = 3. Statistical analysis was performed using a two-way ANOVA followed by Bonferroni’s test. * *p* < 0.05; ** *p* < 0.01; and *** *p* < 0.001.

In 2022, Delta was replaced by Omicron. Figure 4A shows the Omicron survival (BA.2, SARS-CoV-2/human/HKG/Consensus_VOC-588-P3-S18-iseq/2022) compared with Delta on treated tray tables that had been in regular service for 28, 56, and 72 days before removal and testing. Figure 4B shows tray tables that were untreated and treated but in use for 28 days and then removed and inoculated with BA.5 (BA.5, SARS-CoV-2/human/USA/COR-22-063113/2022). Within the first 4 h, the Delta strain had less decrease in titer than BA.2. With BA.2, more virus was detectable after 4 h on the treated tray tables that had been in regular service for 28 and 56 days than treated tables at day 0, but yet again, by 24 h, no virus remained.

### 3.5. Antimicrobial Compound Presence on Treated Surfaces with and without Cleaning

**Figure 5:**
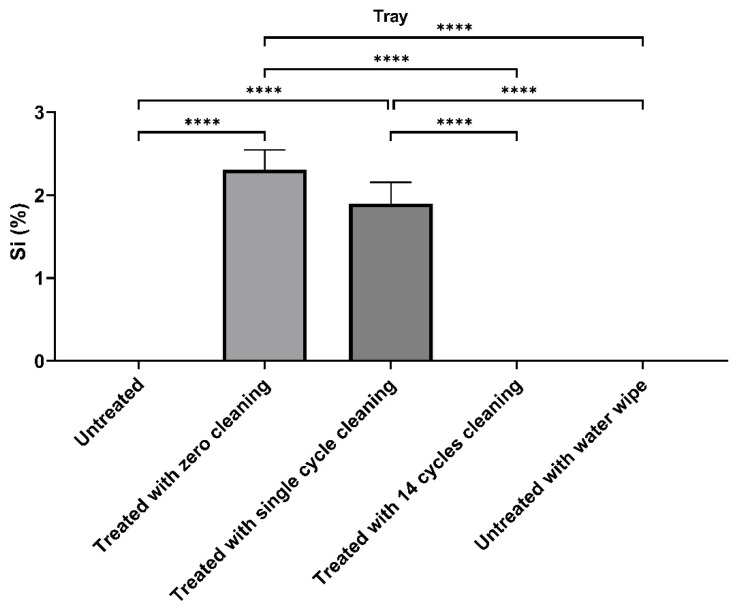
EDS analysis of tray tables that had been electrosprayed with CAS # 27668-52-6. At day 0, the untreated silicon (Si) concentration was 0, after treatment, it was 2.8%. A single cleaning reduced it to 1.7% and after 14 cleaning cycles, the concentration was 0. Data are the mean ± s.d. *n* = 3. Statistical analysis was performed using a one-way ANOVA followed by Bonferroni’s test. **** *p* < 0.0001.

**Figure 6:**
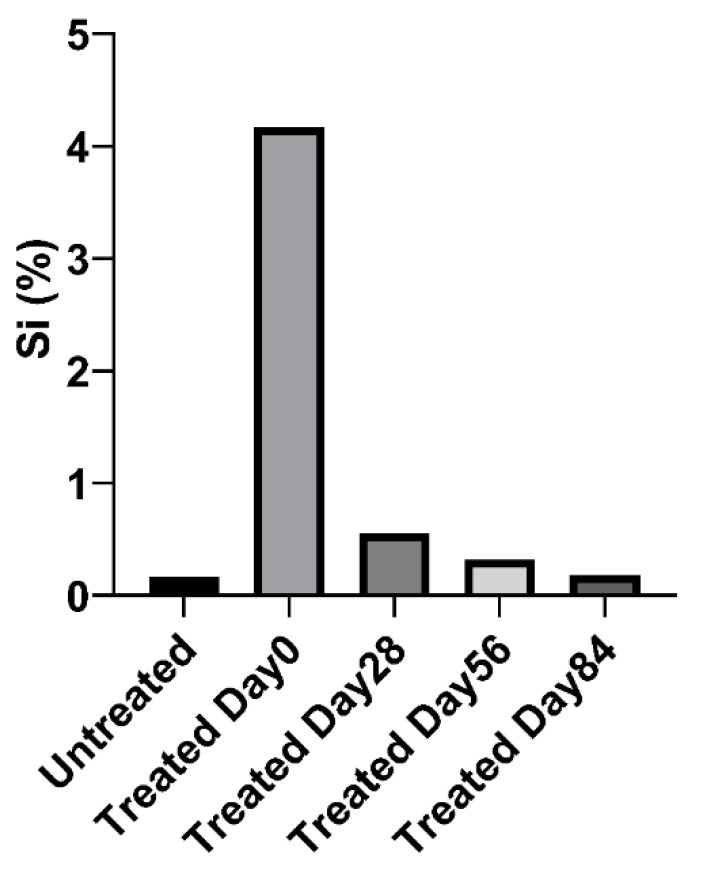
EDS analysis highlighting the silicon (Si) concentration on tray tables after regular service. Si is an indicator of the coating with CAS #27668-52-6. After 28 days of regular use, the level was 15% of Day 1, and after 56 days of regular use, the level was 7% of Day 1.

As the commercial antimicrobial compounds are silicon-based, we used this as a surrogate marker to determine how long the compound would remain on surfaces and investigate how long it could be detected on interior surfaces after regular cleaning with alcohol and other detergent-based compounds, and secondly, how long it would remain on surfaces subject to regular use. Treated surfaces were examined by EDS 24 h after application with a single wipe of domestic cleaners and then after multiple applications. Figure 5 shows that the concentration of silicon decreased from 2.28 to 1.7% after a single cycle of cleaning, and after 14 cycles of treatment in the laboratory setting without any handling, approached baseline. Figure 6 shows that after 28 days of regular service and cleaning, the concentration of silicon decreased by 85%, and in 56 days, decreased by 93% compared to day 0.

## 4. Discussion

This is the first study to address the potential role of the fomite spread of SARS-CoV-2 in the commercial aircraft setting. Our study was conducted to evaluate if the surface environment could play a role in virus survival and/or transmission, as a holistic view is needed to determine if it has a potential role in transmission.

Our previous studies using terrestrial materials (bank notes, paper, and plastic) [12] demonstrated that true infectious viruses could still be cultured for up to two weeks at room temperature, so it was considered important to assess whether a similar result would be seen in aviation material. Since aircraft are normally cleaned within a 24 h period, we focused on stability within the first 24 h of surface inoculation. We noted that there are two important factors that distinguish aircraft interior materials from terrestrial ones. The first is that, in line with regulatory controls, the material must adhere to more stringent fire control parameters, and secondly, apart from the screens of in-flight entertainment, most contact surfaces are not smooth. Viruses have been shown to be more stable on smooth surfaces [32], with less agglomeration which increases their effective life. Furthermore, viruses are more stable on hard rather than porous surfaces and persist longer on hydrophobic materials such as polymers [33].

To decrease virus survival on contact surfaces, the aviation industry has used a number of disinfection strategies. These are quaternary compound-based, and Figure 1, Figure 2, Figure 3 and Figure 4 show that they are effective in reducing virus survival on contact surfaces. As they are silicon-based, we used this as a surrogate marker to determine how long the antimicrobial compound would remain on surfaces and investigated how long it could be detected on interior surfaces after regular cleaning by alcohol and other detergent-based compounds as well as how long it would remain on surfaces subject to regular use. To address the first issue, treated surfaces were examined by EDS 24 h after application with a single wipe of domestic cleaners and then after multiple cycles of application followed by cleaning. Figure 5 shows that the concentration of silicon decreased from 2.28 to 1.7% after a single cycle of cleaning, and after 14 cycles of cleaning in a static laboratory setting, approached baseline. Secondly, after 28 days of regular service and cleaning, the concentration of silicon decreased by 85%, and in 56 days, decreased by 93%, compared to day 0 (Figure 6). This has two important implications for the aviation industry. The first is that electrospraying surfaces does have a significant antimicrobial effect against SARS-CoV-2, but the downside is that regular cleaning will reduce this effectiveness after two cycles. The fact that 15% of the compound could still be detected after 28 days of regular use and with assumed daily cleaning suggests that regular cleaning protocols may need to be improved and closely monitored, and, in the future, EDS (which many airlines have access to) could be used as an indicator of the effectiveness of aircraft cleaning and maintenance.

Figure 6 shows that with regular use and cleaning, the concentration of silicon (Si) decreased from 2.28 to 1.7% and, after 14 treatments, approached baseline. Secondly, after 28 days of regular service and cleaning (Figure 6), the concentration of silicon decreased by 85% but still exerted an antimicrobial effect. This would indicate that if fogging techniques are to be maintained, a 28–56 day period between applications should be adequate.

This study was conducted over a three-year period when there were various “waves” of different SARS-CoV-2 strains. For cost reasons, we were not able to test all surfaces with all the strains of SARS-CoV-2, however, it appears that there has been no major shift in survival on surfaces with successive strains (Figure 4A,B); furthermore, since the wild-type virus and Delta have largely disappeared from the population, more attention should be focused on the current Omicron strains.

The crucial question that this study addressed was whether fomites could play a significant role in the transmission of SARS-CoV-2 or whether the aerosol route was the main mode of transmission [34]. Our previous studies in the early stage of the pandemic raised concerns over the potential role of contact surfaces in transmission, but after three years, consolidated meta-analyses have shown that the evidence for this route is not compelling [35]. However, it should be noted that these meta-analysis studies were mainly conducted when the wearing of face masks was compulsory during travel, which would be a confounding factor.

Our study utilized virus culture, which is acknowledged to be preferable to PCR [36], and demonstrated that, compared to terrestrial daily-use compounds such as banknotes and paper, there was a 1 log (90%) drop within 4 h and little culturable virus present after 24 h. It is important to recognize that our results should not be interpreted as absolute values but viewed in the context of relative change over time; as for experimental consistency, the level of virus input was artificially high [35]. Thus, the more important data are the relative decrease over time. For obvious safety reasons, it is not possible to determine what level of virus can be considered potentially infectious to humans, and limited studies have shown fomite transmission to laboratory animals [37]. There is still debate on whether frozen foods or packaging can contribute to transmission [38,39,40].

## 5. Conclusions

There are three conclusions to our experiments with implications for the aviation industry. The first is that, compared to more porous materials in the terrestrial environment, SARS-CoV-2 is naturally less stable on common contact points in aircraft interiors and that, over a 4 h time period, there will be a 90% reduction in culturable virus, even in untreated samples. This finding raises an interesting question on the inherent difference in virus stability between materials used in the aviation industry and those normally found in terrestrial applications, as the former appears to provide a more hostile environment for virus stability. Future work to understand this difference, such as the incorporation of fire retardants, antimony, or other surface coatings, may lead to reduced transmission by fomites in high-traffic areas used by the general public.

The second conclusion is that, in agreement with published reviews [41], antiviral and surface coatings are extremely effective at mitigating the persistence of viruses on surfaces; however, their benefit is diminished by regular cleaning and will be likely considered to be ineffective after 56 days of regular use. The final point is that successive strains of SARS-CoV-2 have not evolved to be more resilient to survival on aircraft surfaces, and, within the three years of the pandemic, transmission within the confines of an aircraft has been effectively controlled by masks and social distancing. As airline travel has resumed in 2022 and 2023 with fewer requirements for compulsory masks during travel, surface treatment mitigation strategies should not be abandoned as, in addition to inhibiting the survival of SARS-CoV-2, they are effective at reducing the spread of other respiratory and enteric viruses.

## Data Availability

Data are available from the corresponding author upon request.
